# Screening herbal and natural product libraries to aid discovery of novel allosteric modulators of human P2X7

**DOI:** 10.1007/s11302-024-10055-6

**Published:** 2024-10-22

**Authors:** Stefan Bidula, Waraporn Piyasirananda, Hanna Bielecka, Lučka Bibič, Andrew Beekman, Leanne Stokes

**Affiliations:** School of Pharmacy, University of East Anglia, Norwich Research Park, Norwich, NR4 7TJ UK

**Keywords:** Allosteric modulator, P2X7, Scopafungin, Dioscin, Traditional Medicine, Flavonoids, Confertifolin, Digallic acid

## Abstract

P2X7 is an emerging therapeutic target for several disorders and diseases due to its role in inflammatory signalling. This study aimed to exploit the unique chemical libraries of plants used in traditional medicinal practices to discover novel allosteric modulators from natural sources. We identified several compounds from the NCI Natural Product library as P2X7 antagonists including confertifolin and digallic acid (IC_50_ values 3.86 µM and 4.05 µM). We also identified scopafungin as a novel positive allosteric modulator of hP2X7. Screening a traditional medicinal plant extract library revealed 39 plant species with inhibitory action at hP2X7 and 17 plant species with positive allosteric modulator activity. Using computational docking to filter identified components from these plant species and determine potential antagonists, we investigated nine purified chemicals including flavonoids quercetin, kaempferol, ECG, and EGCG. These were shown to inhibit ATP-induced YO-PRO-1 uptake into HEK-hP2X7 cells; however, we also showed that all four flavonoids demonstrated significant assay interference using a cell-free DNA YO-PRO-1 fluorescence test. One plant extract, *Dioscorea nipponica*, demonstrating positive modulator activity was investigated, and dioscin was identified as a glycoside with PAM activity in ATP-induced YO-PRO-1 uptake assay and whole-cell patch-clamp recordings. However, membrane permeabilisation was observed following application > 10 min limiting the use of dioscin as a pharmacological tool. This work describes a useful workflow with multiple assays for the identification of novel allosteric modulators for human P2X7.

**Supplementary Information:**

The online version contains supplementary material available at 10.1007/s11302-024-10055-6.

## Introduction

P2X7 is an ATP-gated ion channel expressed predominantly on immune cells but can also be found more widely in the body including epithelial cells and glial cells in the nervous system [1]. P2X7 plays key roles within inflammation and is likely important in inflammatory disorders whereby it is involved in both canonical and non-canonical NLRP3 inflammasome activation, the release of pro- and anti-inflammatory mediators (e.g. IL-1β, IL-18, annexin A1), shedding of transmembrane proteins (CD62L, CD23), remodelling of the extracellular matrix via release of metalloproteinases, and the regulation of cell death [1, 2]. P2X7 is highlighted as a viable therapeutic target for inflammatory conditions with current emphasis on neuroinflammation, psychiatric conditions, Alzheimer’s disease, and other neurological disorders such as autism and epilepsy [3–6].

Natural products have been highlighted as a rich source of medicinal compounds with many anti-cancer drugs and antibiotics originating from a natural product source [7]. They are often overlooked in preference of synthetic compounds, but they contain therapeutically beneficial molecules which are extremely difficult to synthesise in a laboratory environment. In recent years, a number of positive and negative allosteric modulators for P2X receptors have been discovered from natural products [8–10]. The most widely used of these compounds is the P2X4-positive allosteric modulator (PAM) ivermectin [11] which is chemically derived from *Streptomyces* avermectins. However, the number of allosteric modulators of P2X7 from natural sources is ever-increasing and includes negative allosteric modulators (NAMs) such as caperatic acid, tanshinone II A sulfonate, emodin, and berberine, in addition to PAMs such as ivermectin, isatins, polymyxin B, agelasine, garcinolic acid, and ginsenosides [9, 12–17].

Plant extracts have been utilised for centuries within traditional Chinese medicine (TCM), but investigations into their constituent compounds and the biological effects that these exert are poorly defined. Our laboratory was the first to identify that triterpenoid glycosides, termed ginsenosides from the herb *Panax ginseng*, can positively modulate P2X7-dependent responses [12, 18, 19]. In particular, compound K (CK), the major in vivo protopanaxadiol ginsenoside metabolite, can potentiate ATP-induced channel opening at nanomolar concentrations resulting in enhanced Ca^2+^ flux, macropore formation, and significantly accelerated apoptosis [20]. Conversely, another plant exploited in TCM, *Salvia miltiorrhiza* (Danshen), has been exploited as a source of purinergic receptor antagonists, with tanshinone II A sulfonate antagonising P2X7 [14], salvianolic acid A and C antagonising P2Y1 and P2Y12 receptors, and salvianolic acid B antagonising P2Y12 [21]. Thus, it is likely that other allosteric modulators of purinergic receptors exist within traditional medicinal plants and different approaches exist to investigate this further including a hybrid in silico and docking screen [22].

Our aim in this study was to screen a natural product library (set IV, purified chemicals) and a traditional Chinese medicinal (TCM) crude plant extract library against human P2X7 receptors and identify novel allosteric modulators. Such crude plant extracts likely contain a large number of compounds. We describe a number of potential plant extracts and compounds that can inhibit P2X7, and we characterise the steroidal glycoside dioscin and scopafungin, a compound from *Streptomyces*, which we identify as positive allosteric modulators of P2X7 receptors during short applications. However, dioscin was significantly toxic to cells over time. This paper describes a useful workflow for effective unbiased screening of compounds for P2X7 receptor activity with counter-screens and a novel interference test for the YO-PRO-1 uptake assay.

## Methods

### *Extractsand purified compounds*

The NCI Library of Traditional Chinese Medicinal plant extracts and Natural Product library Set IV were obtained from the Natural Products Branch (NPB), Developmental Therapeutics Program, Division of Cancer Treatment and Diagnosis at the National Cancer Institute (USA). The TCM library was obtained by NCI as a gift from Harvard University through a joint agreement with the NPB and the Office of Cancer Complementary and Alternative Medicine, DCTD, NCI. The origin of source organisms collected for the library has been previously described [23]. Extracts (aqueous and organic) were prepared as previously described [24, 25]. Lyophilised TCM plant extract samples in 96-well plates were reconstituted to 5 mg/mL in sterile DMSO prior to use. The Natural Product library was dissolved in DMSO to 10 mM. The purified compounds of interest were purchased from Chemfaces (Wuhan, China) or Sigma-Aldrich (Poole, UK).

### Cell culture

HEK-293 cells stably transfected with human P2X7 (hP2X7) (EPYME C-terminal tagged hP2X7 in pcDNA3.1 plasmid) or human P2X4 (EPYME-tagged hP2X4 in pcDNA3.1 plasmid) were maintained in DMEM/F-12 media containing L-glutamine (Gibco, ThermoFisher Scientific, catalogue number 11320–074) supplemented with 10% FBS (South American origin, Gibco, catalogue number 10500–064), 100 U/mL penicillin, and 100 µg/mL streptomycin (Life Technologies, Fisher Scientific) with continual selection under 400 µg/mL G418 (Life Technologies, Fisher Scientific). Human THP-1 monocytes (obtained from Professor Maria O’Connell, University of East Anglia) were maintained in RPMI-1640 media containing L-glutamine (Gibco, ThermoFisher Scientific, catalogue number 21875–034) supplemented with 10% foetal bovine serum (Sigma-Aldrich, US origin, catalogue number F2442) and 100 U/mL penicillin plus 100 µg/mL streptomycin. All cells were maintained at 37 °C in a 5% CO_2_ humidified incubator.

### Dye uptake experiments

HEK-hP2X7 or HEK-hP2X4 cells were plated at 2.5 × 10^4^ cells per well (100 µL volume) the day before experiments into poly-D-lysine (Merck Millipore) coated 96-well plates (NUNC, Fisher Scientific #167-008). The membrane impermeant dye YO-PRO-1 iodide (Life Technologies, Fisher Scientific) was used at a final concentration of 2 µM in low divalent buffer (145 mM NaCl, 5 mM KCl, 0.2 mM CaCl_2_, 13 mM glucose, 10 mM HEPES, pH 7.3, osm 300–310). Media were removed from the plate, and 90 µL of YO-PRO-1 buffer was added per well. Plates were warmed to 37 °C for 5 min before recording. Data was acquired on a Flexstation 3 plate reader (Molecular Devices) using excitation wavelength 490 nm, emission wavelength 520 nm, and six reads per well (PMT medium). Compounds or plant extracts at 10X final concentration were automatically injected at 30 s, and then, ATP was automatically injected at 90 s from a 96-well V-bottomed drug plate (Greiner). This enabled cells to be pre-treated with compounds/plant extracts for 60 s before the agonist was injected. Pipette height was set to 80 µL and the rate of injection was 4. Data was analysed as AUC between 100 and 300 s using SoftMax Pro v5.4 software (Molecular Devices).

A cell-free assay was developed as a counter-screen for compound interference with the YO-PRO-1 dye uptake cellular assay. YO-PRO-1 iodide was used at 2 µM in a low divalent buffer (see above). Drugs were made at final concentration in this YO-PRO-1 assay buffer, and 90 µL was added per well of a 96-well plate. Plates were warmed to 37 °C for 5 min before recording. Calf thymus DNA (Thermo Fisher) was diluted over the range of 100 µg/mL to 100 pg/mL in low divalent assay buffer. Ten microlitres of each DNA concentration was automatically injected into wells using the Flexstation 3 fluidic function, and fluorescence increase was monitored for 60 s. The same recording settings were used as for the cellular assay, excitation wavelength 490 nm, emission wavelength 520 nm, and six reads per well.

### Cell viability assays

Cells were plated at 2 × 10^4^ cells/well in Nunc 96-well plates (Fisher Scientific #167-008) 24 h prior to stimulation with ATP 500 µM or 3 mM in combination with identified positive or negative crude plant extracts, respectively, or with purified compounds (10 µM) for 24 h. Following incubation, resazurin (0.1 mg/mL in PBS, Sigma-Aldrich) was added to cells for 2 h at 37 °C and fluorescence was measured on a Flexstation 3 plate reader (excitation 570 nm; emission 600 nm).

### IL-1β ELISA

IL-1β concentrations were quantified using a BD OptiEIA™ human IL-1β kit (BD Biosciences). In brief, THP-1 cells were treated with LPS for 4 h, prior to the addition of ATP (3 mM) for 30 min. To investigate the effect of negative allosteric modulators, compounds were added at the same time as LPS and incubated for 4 h. To investigate the effect of the positive allosteric modulators, compounds were added with the ATP following 4 h of LPS stimulation. Following stimulation, cells were centrifuged at 3000 g for 5 min, and supernatants were transferred to a new Eppendorf and frozen at − 80 °C. IL-1β was measured in supernatants as per the manufacturer’s instructions using a Flexstation 3 plate reader to read absorbance at 450 nm.

### Patch-clamp electrophysiology

Stably expressing HEK-hP2X7 cells were plated onto 13-mm glass coverslips 24 h prior to use. Membrane currents were recorded in the whole-cell patch-clamp configuration using an EPC10 amplifier (HEKA Elektronik) and borosilicate glass electrodes (TF-150 World Precision Instruments), resistance 3–8 Ω when filled with standard internal solution (145 mM NaCl, 10 mM HEPES, 10 mM EGTA, pH 7.3). Cells were voltage-clamped at − 60 mV, and dialysis of intracellular contents was performed for 1 min prior to experimental procedures. Cells were continuously perfused by gravity feed with standard divalent buffer solution (145 mM NaCl, 5 mM KCl, 2 mM CaCl_2_, 1 mM MgCl_2_, 13 mM glucose, 10 mM HEPES, pH 7.3) prior to seal formation, and low divalent buffer solution was used for agonist and PAM application. Agonists were applied using a computer-controlled fast-flow system (RSC-160; Bio-Logic Scientific Instruments) with the perfusion capillaries placed in close proximity to the cell under investigation.

### Computational docking

The hP2X7 homology model was generated as previously described [18]. The CADD Group Chemoinformatics Tools and User Services (CACTUS) online SMILES translator and structure file generator were used to generate PDB files of 157 potential compounds found in the TCM extracts. Preliminary docking of these molecules to the negative allosteric site on hP2X7 was performed using a cubic box with dimensions of 22–26 Å in AutoDock Vina [26]. For induced fit docking, ligands were prepared virtually using LigPrep within the Schrodinger Maestro software. The OPLS3 forcefield was used to generate conformers for each ligand, and induced fit docking was performed using Glide as previously described [18]. The receptor grid was centred on the predicted positive allosteric site of P2X7 [18]. Final ligand–protein complexes within 30 kcal/mol of the lowest energy structure were retained. The resulting poses were clustered by heavy atom RMSD using the average-linkage method, and a representative structure was chosen from the model closest to the centroid of the most populated cluster.

### Data analysis

YO-PRO-1 dye uptake assay data is plotted as a percentage of control where control responses are measured in the absence of plant extract. Cell viability data is plotted as a percentage of control or background-corrected fluorescence data. Concentration–response graphs were plotted using GraphPad Prism v6 with a non-linear regression fit (variable slope). IC_50_ values were generated from collated datasets from a minimum of three independent experiments. Statistical differences were determined by one-way ANOVA statistical tests performed using GraphPad Prism v6 with Dunnett’s multiple comparison post hoc test. Significance was taken as *P* ≤ 0.05.

## Results

### Screening of a natural product library Set IV for modulators of human P2X7

We acquired a natural product library (Set IV) containing 419 compounds from NCI DTP and tested these on P2X7 responses using a YO-PRO-1 dye uptake in HEK293 cells stably expressing human P2X7 (HEK-hP2X7). This screen used a two-injection procedure on a Flexstation 3 plate reader, whereby the compounds (at a fixed concentration of 10 µM) were injected first, and 60 s later, the agonist ATP was injected, similar to the method employed for screening crude spider venoms [27]. This allowed us to assess for any immediate effect of the injected compound, and secondly, it allowed for any potential negative modulators to be pre-incubated with P2X7. Our criteria for identifying potential positive modulators were compounds increasing the ATP response by > 25%, and such compounds are listed in Supplementary Table 1. Conversely, any potential negative modulators were defined as any compounds that reduced the YO-PRO-1 uptake by > 50% (Supplementary Table 2). We excluded compounds that had shown an immediate increase in YO-PRO-1 fluorescence upon injection as this indicated immediate cell toxicity.

**Fig. 1:**
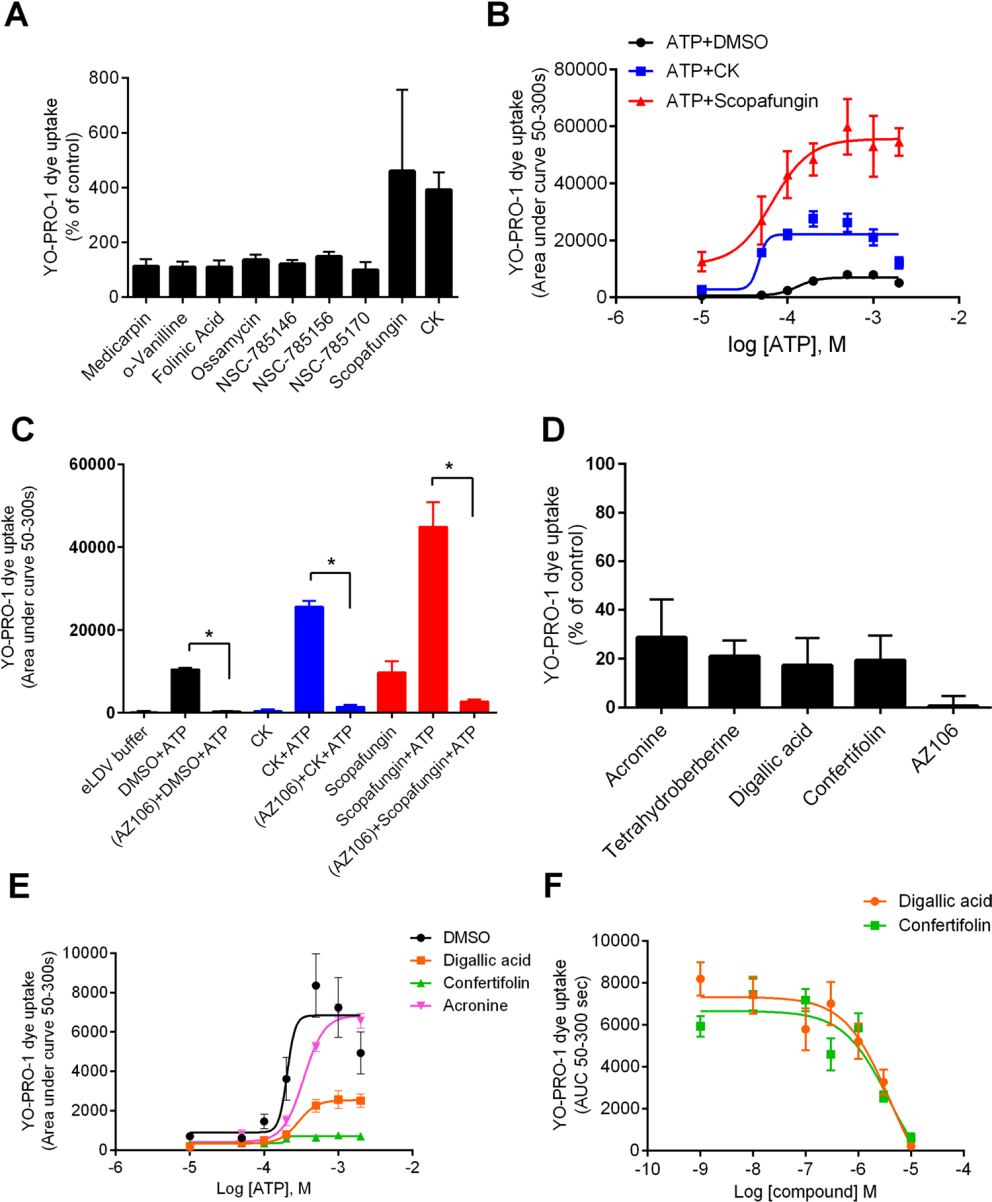
Purified Natural Product Set IV chemicals with action at hP2X7. **A** HEK-hP2X7 cells were pre-treated with diversity set IV compounds or ginsenoside CK (10 µM) for 10 min. ATP-dependent YO-PRO-1 iodide dye uptake was measured over 300 s following administration of ATP (1 mM). **B** Concentration–response curve for ATP in the presence of ginsenoside CK (10 µM) or scopafungin (10 µM) using the YO-PRO-1 dye uptake assay. **C** Inhibitory effect of AZ10606120 (10 µM) on responses elicited by ATP in the presence of either CK or scopafungin. **D** HEK-hP2X7 cells were pre-treated with diversity set IV compounds or AZ10606120 (10 µM) for 10 min. ATP-dependent YO-PRO-1 iodide dye uptake was measured over 300 s following administration of ATP (1 mM). **E** Concentration–response curve for ATP in the presence of digallic acid, confertifolin, or acronine (10 µM) using the YO-PRO-1 dye uptake assay. **F** Dose inhibition curve for confertifolin and digallic acid at hP2X7. The symbol “*” represents statistical significance (*P* < 0.05) from one-way ANOVA with Sidak’s multiple comparison post hoc test

We first investigated potential PAMs by re-testing several selected compounds in a co-injection experiment (Fig. 1A). The compound showing the largest potentiating effect was scopafungin, and we investigated whether it would cause a leftward shift in the ATP concentration–response curve. Figure 1B shows that scopafungin could increase the maximum response to ATP and increase the sensitivity to ATP; the EC_50_ to ATP (DMSO) was 131.3 µM (95% confidence interval 95.8 to 180 µM), whereas in the presence of scopafungin, this was reduced to 66.8 µM (95% confidence interval 2.9 to 151.6 µM). We considered that scopafungin could be a non-specific compound similar to solanine and saikosaponin A from our previous study [28]. Scopafungin did cause an increase in YO-PRO-1 uptake in the absence of ATP; however, the response induced by ATP plus scopafungin could be prevented by pre-treatment with the P2X7 selective antagonist, AZ10606120 (Fig. 1C), suggesting that some of its activity could be P2X7-dependent. This compound could be further studied as a potential new PAM at P2X7; however, its large molecular weight and complex structure make this an unlikely lead molecule for future development.

Investigations into potential negative allosteric modulators revealed 19 compounds (Supplementary Table 2) meeting the screening criteria. We investigated several of these compounds in further experiments using a longer pre-incubation time (10 min) in the YO-PRO-1 dye uptake assay. Acronine, confertifolin, digallic acid, and tetrahydroberberine (10 µM) were all able to reduce ATP-mediated YO-PRO-1 uptake (Fig. 1D). Other compounds with potential NAM activity (Supplementary Fig. 2) were not confirmed in this study. We also tested several compounds on the ATP concentration–response curve (Fig. 1E) revealing a potential non-competitive mechanism of action for digallic acid and confertifolin. Dose inhibition experiments calculated the IC_50_ values for confertifolin and digallic acid to be 3.86 µM and 4.05 µM respectively. These may be interesting lead molecules for future antagonist development.

### Screening of a traditional Chinese medicine (TCM) plant extract library revealed both positive and negative modulators of P2X7

**Fig. 2:**
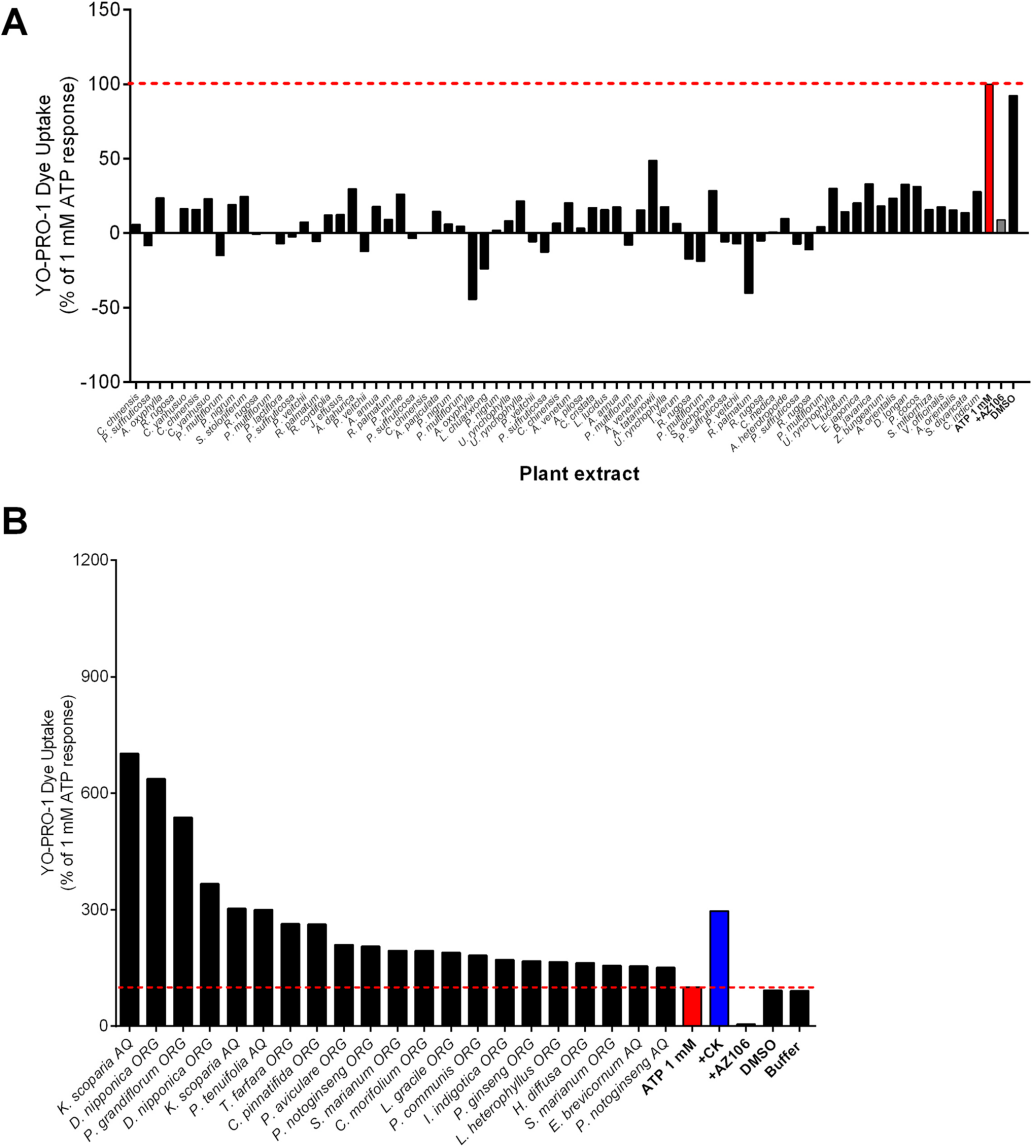
Extracts exhibiting inhibition or potentiation of hP2X7. HEK-hP2X7 cells were pre-treated with 30 µg/mL of TCM extract for 60 s prior to the administration of 1 mM ATP. YO-PRO-1 uptake was measured for an additional 210 s, and the YO-PRO-1 uptake between 200 and 300 s was quantified as a percentage of the ATP response in the absence of extracts. The addition of 10 µM AZ10606120 was added as a control for inhibition, 10 µM CK was used as a control for potentiation, and DMSO was used as a vehicle control. **A** Extracts exhibiting more than 75% inhibition were considered inhibitory. **B** Extracts enhancing ATP responses to > 150% of the original ATP response were considered potentiators. Data are representative of two technical replicates but one independent experiment due to the availability of the extracts. Red line indicates the control response in each case

In the second part of this study, we screened organic and aqueous plant extracts that are commonly used in traditional Chinese medicine practices to identify novel compounds that could allosterically modulate hP2X7. We screened almost 400 extracts, the identity of which was unknown to us at the time of testing allowing for an unbiased approach to screening. Again, we used a two-injection procedure in a Flexstation 3 plate reader, whereby the crude extracts were injected first (to a final concentration of 30 µg/mL), and 60 s later, the agonist ATP was injected. Our criteria for identifying potential negative modulators were extracts reducing the YO-PRO-1 uptake by > 75% (Fig. 2A), and 71 extracts from 39 identified plant species meeting these criteria are listed in Supplementary Table 3. Conversely, positive modulators were identified as extracts increasing the ATP response to > 150% of control (Fig. 2B), and 29 extracts from 17 plant species meeting these criteria are detailed in Supplementary Table 4. We next confirmed the activity of these plant extracts by performing further blinded tests using alternative assays for hP2X7 activity, namely an IL-1β secretion assay on LPS-primed THP-1 monocytes and a cell viability assay on HEK-hP2X7 cells. We treated THP-1 monocytes with LPS (100 ng/mL) together with the crude plant extracts for 4 h, prior to stimulation of P2X7 responses with 3 mM ATP for the final 30 min. The data indicated that all but two of the extracts (*Sparganium stoloniferum* and *Prunus mume*) also had inhibitory effects on the secretion of IL-1β following ATP stimulation (Supplementary Fig. 1A). Another physiological function linked to the activation of P2X7 is the induction of cell death pathways [20]. P2X7 activation has been linked to cell death via numerous pathways including apoptosis, pyroptosis, and necrosis [29]. Using HEK-hP2X7 cells, we pre-treated the cells with 30 µg/mL of the inhibitory plant extracts (1 h) and added a lethal concentration of ATP (3 mM). Cells were assessed for viability using the AlamarBlue metabolic assay after 24 h (Supplementary Fig. 1B). Treatment with AZ10606120, a selective P2X7 antagonist, served as the control for this experiment. Of the 71 extracts tested, 51 were observed to rescue ATP-induced cell death by ≥ 50% (Supplementary Fig. 1) and 20 plant extracts exhibited inhibition comparable to AZ10606120. All results were then collated and submitted to the NCI DTP office to release the identities of the plant extracts we considered to be hits. These identified inhibitory hit extracts were further tested for specificity against hP2X7 by counter-screening against HEK-293 cells expressing human P2X4 using the YO-PRO-1 dye uptake assay. Only seven extracts from plants *Polygonum multiflorum*, *Paeonia suffruticosa*, *Rosa rugosa*, *Rheum palmatum*, *Paeonia lactiflora*, *Paeonia veitchii*, and *Andrographis paniculata* also showed an inhibitory effect against this receptor (Supplementary Fig. 2). This data could be useful for identifying compounds with inhibitory activity against hP2X4.

### Compounds contained in the inhibitory plant extracts are predicted to bind to the negative allosteric site on P2X7

**Fig. 3:**
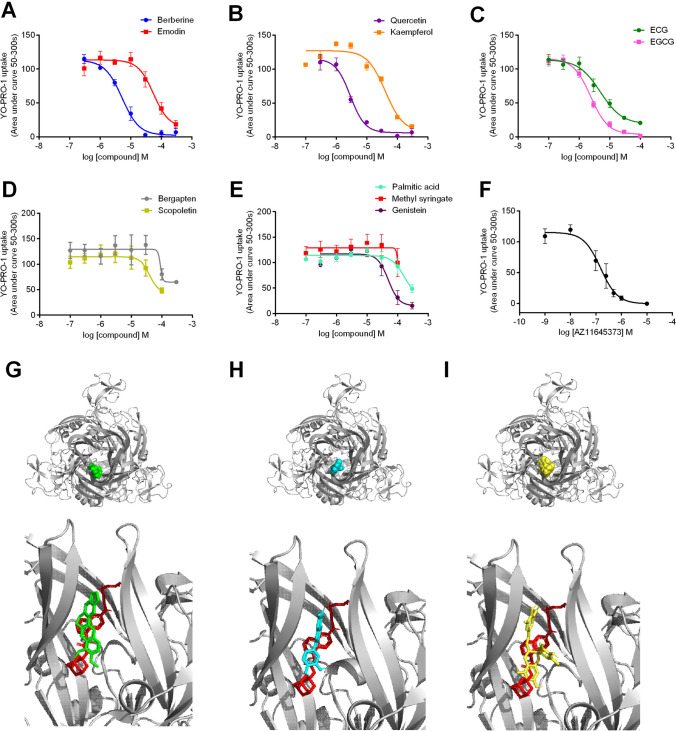
Inhibitory effect of purified chemicals on hP2X7 responses. HEK-hP2X7 cells were pre-treated with purified compounds for 10 min, and ATP-dependent YO-PRO-1 iodide dye uptake was measured over 300 s following administration of ATP (1 mM). Concentration–response curve for **A** berberine and emodin; **B** quercetin and kaempferol; **C** ECG and EGCG; **D** bergapten and scopoletin; **E** palmitic acid, methyl syringate, and genistein; and **F** AZ11645373. Induced fit docking for **G** berberine (green), **H** quercetin (cyan), and **I** EGCG (yellow) at hP2X7 NAM site using a homology model built on zfP2X4. AZ10606120 is shown in red as a comparison

Plant extracts that showed inhibitory activity in all three assays (P2X7-dependent YO-PRO-1 uptake, IL-1β secretion, and cell death) (Supplementary Table 3) were taken to be true hits and were taken forward for further investigation. A basic (but not exhaustive) search of plant catalogues identified 157 distinct compounds that could be found within these inhibitory extracts. The SMILES strings for each of these compounds were obtained from PubChem and converted into a ligand library using CACTUS prior to computational docking. A single negative allosteric modulator site on P2X7 has been well characterised and is able to accommodate many diverse P2X7 antagonists [29]. Preliminary docking using AutoDock Vina software highlighted 59 compounds to be capable of interacting with this region, many of which were chemically distinct (Supplementary Table 5). These compounds included the known P2X7 inhibitors emodin (known to be found in *P. multiflorum*, *R. palmatum*, and *A. tatarinowii*) and berberine (known to be found in *C. yanhusuo* and *C. chinensis*), but also several other compounds such as flavonoids kaempferol, quercetin, genistein, epicatechin gallate (ECG), epigallocatechin gallate (EGCG), and bergapten, methyl syringate, scopoletin, and palmitic acid. We purchased these nine commercially available purified compounds to further investigate their ability to inhibit P2X7-dependent YO-PRO-1 uptake in HEK-hP2X7 cells. Berberine, quercetin, ECG, and EGCG showed inhibition of ATP-induced YO-PRO-1 uptake with IC_50_ values less than 10 µM (Table 1). Several of the purified compounds (scopoletin, palmitic acid, bergapten, and genistein) showed a reduction of YO-PRO-1 uptake in hP2X7 cells at high concentrations (> 30 µM) (Fig. 3D, E). Induced fit docking of these compounds was then performed on a human P2X7 homology model confirming potential interactions with the hP2X7 NAM site (Fig. 3).

**Table 1:** IC_50_ values for potential negative allosteric modulators at hP2X7 from YO-PRO-1 dye uptake assay; *nc*, not calculated

Compound	IC_50_ value (95% CI)
AZ11645373	152.9 nM (81.7–286 nM)
Berberine	5.17 µM (3.73–7.18 µM)
Quercetin	2.91 µM (2.1–4.01 µM)
ECG	4.96 µM (2.81–8.74 µM)
EGCG	2.39 µM (1.84–3.11 µM)
Kampferol	40.0 µM (25.2–63.5 µM)
Emodin	55.2 µM (29.6–102.8 µM)
Genistein	49.7 µM (27.0–91.6 µM)
Palmitic acid	166.8 µM (1.6–16.9 mM)
Methyl syringate	nc
Bergapten	nc
Scopoletin	39.9 µM (1.25–123.8 µM)

**Fig. 4:**
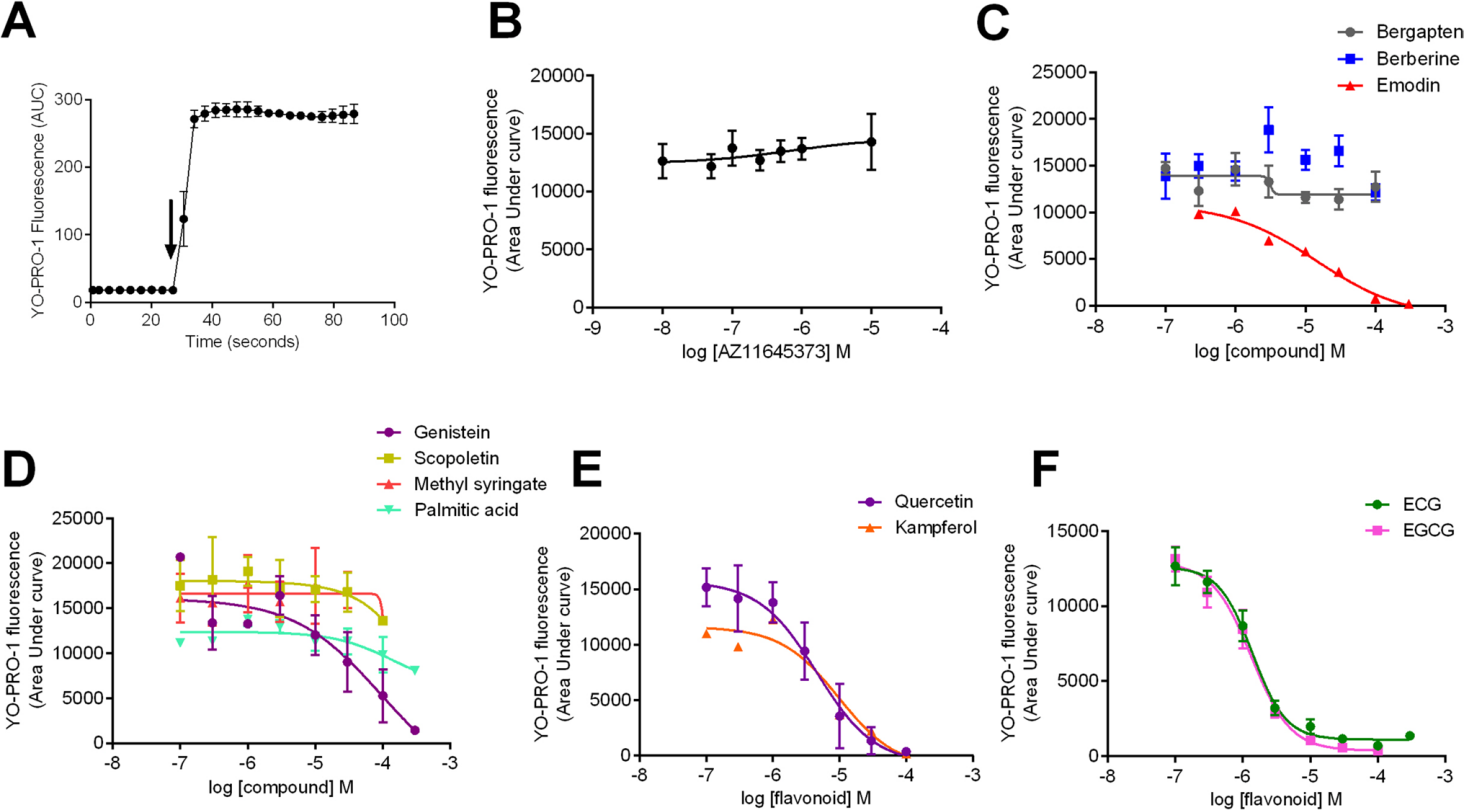
Cell-free YO-PRO-1 assay to measure compounds with interference. Compounds were diluted in a low divalent cation buffer containing 2 µM YO-PRO-1 iodide and 90 µL plated per well. Calf thymus DNA (1 µg/mL) was injected at 30 s using a Flexstation 3 and fluorescence measured over a further 60 s. **A** A typical response to DNA under control conditions. **B** Lack of interference by AZ11645373. Dose–response curves show the effects of **C** bergapten, berberine, and emodin; **D** genistein, palmitic acid, scopoletin, and methyl syringate; **E** quercetin and kaempferol; and **F** ECG and EGCG over the concentration range 100 nM–100 µM

We were concerned that the identified flavonoid compounds could be interfering with the YO-PRO-1 fluorescence assay [30, 31], and therefore, we designed a cell-free interference assay to determine if this was the case. The entry of the YO-PRO-1 dye into the cytoplasmic space of cells enables binding to nucleic acids such as RNA and DNA which causes a measurable increase in dye fluorescence. Therefore, any interference of the ability of YO-PRO-1 dye to interact with cellular DNA/RNA would reduce the measured fluorescence. In the cellular assay, the compounds are pre-treated with cells in a buffer containing YO-PRO-1; therefore, compounds could interact with YO-PRO-1 dye directly or could enter cells and interact with cellular nucleic acids to act as interfering agents. The cell-free assay was designed to inject a known concentration of DNA into the YO-PRO-1 buffer and measure the resulting fluorescence (Fig. 4A). AZ11645373 has no interfering effect on YO-PRO-1 fluorescence (Fig. 4B); however, some of the flavonoids can be seen to inhibit YO-PRO-1 fluorescence, suggesting that these could be interacting with DNA or quenching dye fluorescence (Fig. 4). Quercetin, kaempferol, ECG, EGCG, emodin, and genistein all displayed interference with the assay (Fig. 4). Bergapten, berberine, scopoletin, and methyl syringate demonstrated no interference. Quercetin was tested on ATP-induced calcium responses using fura-2-loaded HEK-hP2X7 cells, and this data further supports a lack of real P2X7 antagonist activity (Supplementary Fig. 3), whereas EGCG and berberine both demonstrated P2X7 antagonist activity in this assay.

### Plant extracts identified with potentiator activity can enhance P2X7-dependent IL-1β release and enhance P2X7-mediated cell death

**Fig. 5:**
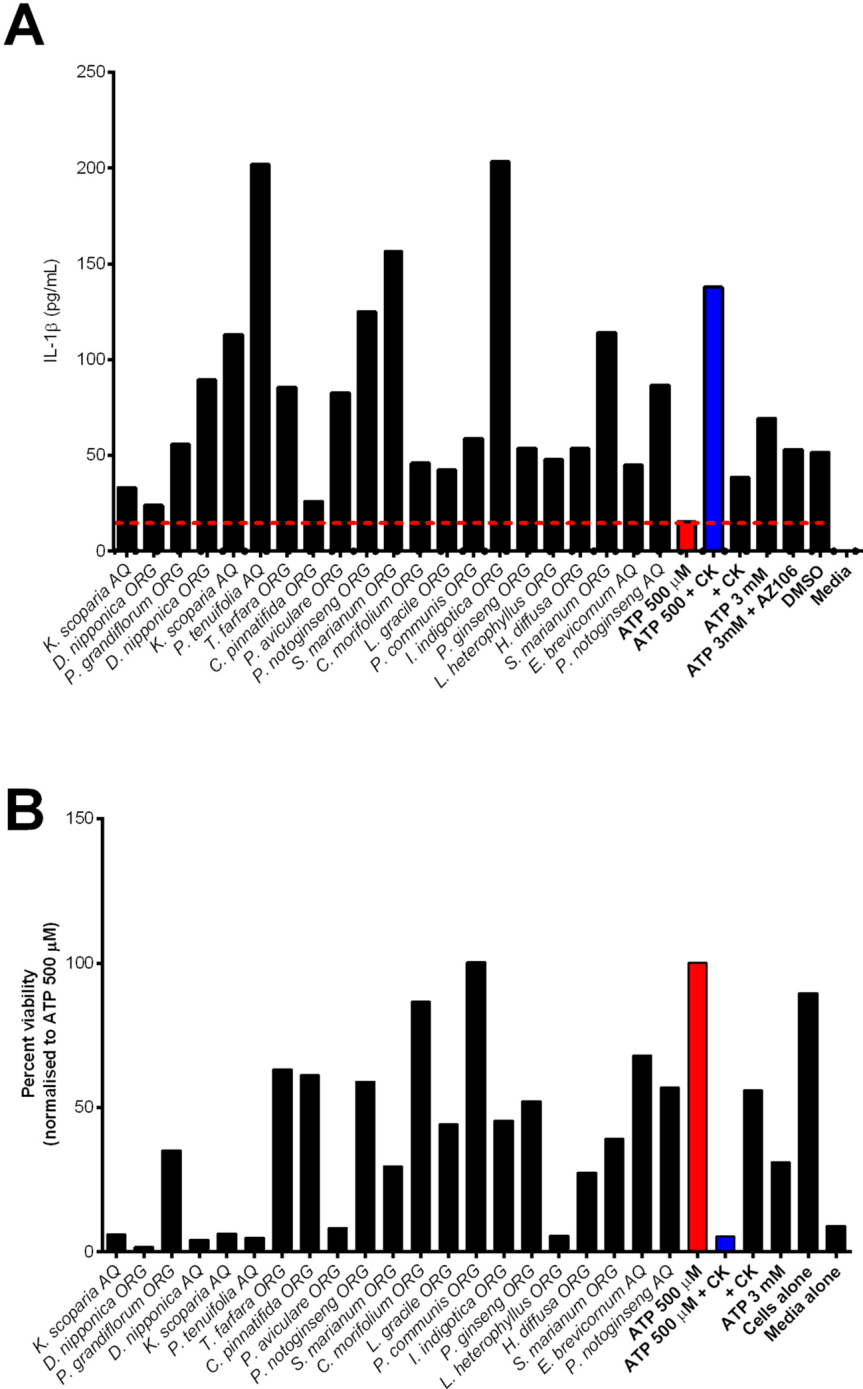
Plant extracts that potentiate hP2X7 dye uptake responses can enhance the secretion of IL-1β and cell death responses. **A** THP-1 cells were treated with LPS (100 ng/mL) for 4 h followed by the addition of TCM plant extract (30 µg/mL) and ATP (500 µM) for the final 30 min of incubation. IL-1β release into supernatants was quantified and compared to the secretion of IL-1β in the absence of plant extract. Ginsenoside CK (10 µM) was used as the positive control and 3 mM ATP was used as a control to demonstrate P2X7-induced IL-1β secretion. **B** HEK-hP2X7 cells were treated with ATP (500 µM) in the presence or absence of selected TCM plant extracts (30 µg/mL) for 24 h prior to quantification of viable cells by using AlamarBlue assay. Ginsenoside CK (10 µM) was used as a positive control for the enhancement of cell death. Data are representative of two technical replicates from one independent experiment due to the limited availability of the TCM plant extracts

We next focused our investigation on the crude TCM plant extracts that could potentiate P2X7-mediated responses. Those extracts that stimulated YO-PRO-1 responses themselves, or gave variable effects, were removed from further experiments. The extracts were ranked from greatest potentiating effects to the least potentiating effects (Fig. 2B). To date, the most effective PAM of P2X7 we have identified is the ginsenoside compound K (CK) [12, 18, 28], which we used here as a positive control. From the screen, we identified crude extracts from four other plants that could increase the hP2X7 response more than ginsenoside CK (Fig. 2B), from plants *Kochia scoparia*, *Dioscorea nipponica*, *Platycodon grandiflorum*, and *Polygala tenuifolia*. Some of the identified plant extracts that potentiated P2X7 responses were from *Panax notoginseng* and *Panax ginseng*, which contain compounds we already know act as PAMs on P2X7 [12], and this was an important unbiased internal control. We first confirmed the effects of the PAMs on the potentiation of P2X7-dependent IL-1β secretion. THP-1 cells were stimulated for 4 h with LPS, prior to stimulation with 500 µM ATP and the respective plant extract for the final 30 min of the incubation. This resulted in some variability, and unexpectedly, the greatest potentiators identified in the YO-PRO-1 assay did not stimulate the greatest IL-1β release. Instead, *Isatis indigotica* elicited the greatest IL-1β response, followed by *P. tenuifolia*, *P. notoginseng*, *and S. marianum* (Fig. 5A). Extracts of *Kochia scoparia* and *Dioscorea nipponica* were capable of potentiating ATP-induced IL-1β release (Fig. 5A). We then tested whether the PAMs could potentiate the cell death of HEK-hP2X7 cells exposed to a non-lethal concentration of ATP (500 µM) after a 24-h incubation [28]. Under these conditions, extracts from *K. scoparia*, *D. nipponica*, *P. tenuifolia*, *T. farfara*, *P. aviculare*, and *L. heterophyllus* enhanced the killing of HEK-hP2X7 cells to a level comparable to the positive control, ginsenoside CK (Fig. 5B). Extracts from *C. morifolium* and *P. communis* were the only hit extracts to not enhance cell death in these experiments (Fig. 5B). However, it must be kept in mind that there was high variability therefore meaningful conclusions due to the data being from one experiment due to a limited sample material (Fig. 5B). Using our counter-screen against the related receptor hP2X4, none of the extracts that had a positive modulatory effect at P2X7 had a positive modulator effect at P2X4 (Supplementary Fig. 2).

### Dioscin, a compound found in *D. nipponica* is a potential PAM for P2X7

**Fig. 6:**
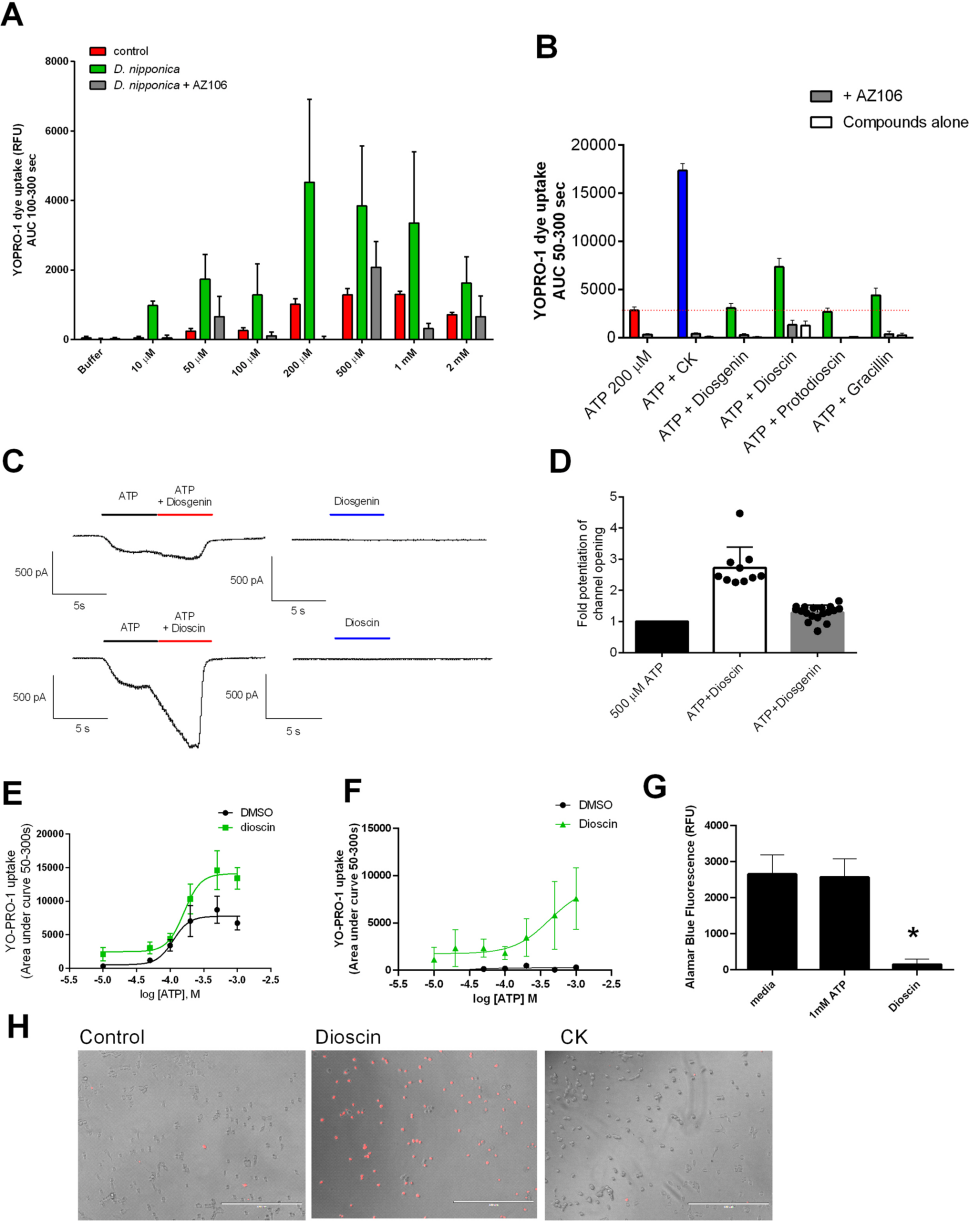
Dioscin enhances P2X7-dependent pore formation and potentiates channel opening. **A** Dioscorea nipponica (organic) plant extract (30 µg/mL) increased ATP-dependent YO-PRO-1 iodide uptake. AZ10606120 (10 µM, grey bars) was added to block hP2X7. **B** YO-PRO-1 uptake following a co-stimulation of HEK-hP2X7 cells with 200 µM ATP ± the named compounds in the presence or absence of AZ10606120. YO-PRO-1 uptake was quantified between 50 and 300 s (*n* = 5). **C** Whole-cell patch-clamp recordings were performed at room temperature. Cells were voltage-clamped at − 60 mV, and responses to a 5-s stimulation of ATP (100 µM) followed by a 5-s stimulation of ATP (100 µM) + dioscin/diosgenin (10 µM) were measured from HEK-hP2X7 cells. Representative traces are shown. **D** Summary of normalised current amplitudes represented as a fold change compared to ATP responses in the absence of dioscin/diosgenin (*n* = 10–18 cells). **E** Concentration response to ATP in the presence of dioscin (10 µM, green) or vehicle (DMSO, black) using YO-PRO-1 uptake assay in HEK-hP2X7 and **F** HEK-293 cells. **G** Cell viability assay using AlamarBlue shows dioscin (10 µM) is toxic over 24 h in HEK-293 cells. 1 mM ATP does not induce cell death. **H** HEK-293 cells were treated with dioscin or CK (10 µM) for 10 min and stained with propidium iodide to reveal permeabilised cells. Images taken with EVOS microscope

From the screening results, we researched the crude plant extracts to identify compounds as active PAMs at P2X7. We chose to investigate the *D. nipponica (organic)* extracts further since this plant contained several glycosides with structural similarity to the ginsenosides (dioscin, protodioscin, and gracillin), and we tested whether a fixed concentration of extract (10 µg/mL) could potentiate ATP responses in a P2X7-dependent manner. *D. nipponica* extract could potentiate all concentrations of ATP tested, and this response could be inhibited by the selective P2X7 antagonist AZ10606120 (Fig. 6A). We previously identified that protopanaxadiol glycosides from *P. ginseng* are PAMs for P2X7 [12, 28], and for this reason, glycosides dioscin, protodioscin, and gracillin and the diosgenin aglycone (control) were docked against P2X7. Dioscin was predicted to bind to a site previously described to be involved in positive allosteric modulation (Supplementary Fig. 4). Diosgenin and protodioscin were not predicted to interact with this site at all, and gracillin was not predicted to make polar interactions at this site. We tested the ability of these purified glycosides to potentiate P2X7-dependent dye uptake responses in HEK-hP2X7 cells. In the presence of 200 µM ATP (approx. EC_50_ value), dioscin was able to enhance YO-PRO-1 dye uptake and this could be reduced with AZ10606120, indicating the involvement of P2X7 in the response (Fig. 6B). Whole-cell patch-clamp electrophysiology confirmed that dioscin could potentiate hP2X7 ion channel opening and that dioscin alone was not inducing ion channel opening itself during the short 5-s application (Fig. 6C). The aglycone diosgenin was not capable of potentiating P2X7 channel opening (Fig. 6C, D). Exploring the type of PAM activity at hP2X7, we performed a dose response to ATP in the absence/presence of 10 µM dioscin using the YO-PRO-1 assay and observed an increase in the maximum response with no leftward shift and no major effect on the EC_50_ value for ATP (110 µM vs 160 µM in the presence of dioscin) (Fig. 6E). However, dioscin could also increase YO-PRO-1 dye uptake in non-transfected HEK-293 cells in the presence of ATP (Fig. 6F) suggesting a non-specific effect. We investigated whether this was due to cytotoxicity. Dioscin could induce cell death in non-transfected HEK-293 cells over 24 h (Fig. 6G), and dioscin was able to induce propidium iodide staining of HEK293 cells within 10 min of addition (Fig. 6H) suggesting a non-specific membrane permeabilisation and cytotoxic action.

## Discussion

This study utilised a high-throughput fluorescent screening method in combination with validation and interference assays plus molecular modelling to unveil novel modulators of P2X7 from a natural product library and plant extracts used in traditional Chinese medicine. Screening in this manner resulted in the identification of a number of hit plant species with pharmacological activity at hP2X7, and our detailed methodological approach could be extremely useful for others to follow this P2X7 pipeline for discovery of novel modulators. A similar system could be adapted for P2X4 drug discovery, and we have previously established a similar methodological approach for studying venom-related molecules [27].

In this study, our primary aim was to pursue positive modulators of P2X7, but our experiments also unveiled several inhibitory extracts. We identified extracts as NAMs if they were capable of inhibiting P2X7-dependent YO-PRO-1 dye uptake, plus showed inhibitory activity in P2X7-dependent cell responses such as cell death and the production of IL-1β. Initially, we chose to focus on the compounds that were predicted to bind to a known inhibitory NAM site within the extracellular domain of P2X7, as this site has been extensively characterised by site-directed mutagenesis and X-ray crystallography studies [29, 32]. Notably, most compounds that were predicted to interact with this site were chemically distinct and included flavonoids, phenols, fatty acids, phenylpropanoids, monoterpenoids, furans, acids, alkaloids, triterpenoids, lignans, coumarins, iridoids, and sesquiterpenoids (Supplementary Table 5). Although the sheer chemical diversity of compounds predicted to bind to this region was unexpected, the known antagonists for this site are also very chemically diverse [33]. We chose to study purified chemicals after using literature sources to identify likely active constituents, and we found that several flavonoid compounds were inhibitory in P2X7 dye uptake assays. These showed similar activity to berberine, an alkaloid already shown to have P2X7 antagonist activity [15, 34] and the anthraquinone emodin [16, 35]. No full pharmacological characterisation for berberine at human P2X7 could be found to compare our IC_50_ value. Emodin was not as effective at inhibiting HEK-hP2X7 responses in our assay as previously reported by others; in YO-PRO-1 uptake experiments, the IC_50_ for emodin was 55.2 µM at hP2X7 (Table 1), whereas an IC_50_ of 3 µM was seen by others when using electrophysiological recordings [16]. However, we also observed some interference in the YO-PRO-1 assay using emodin (Fig. 4) as determined by a new cell-free method developed in this study. Many of the flavonoid compounds were found to interfere with the YO-PRO-1-DNA assay; this is likely either by interacting with the dye molecule, interacting with DNA, or preventing the dye from intercalating with DNA. Flavonoids such as quercetin and catechins have been shown to bind to DNA [36, 37]; therefore, this might be the source of the interference in this assay. In the cellular YO-PRO-1 assay, flavonoids could be taken into the cytoplasm where they could interact with DNA/RNA molecules preventing the dye from interacting and producing fluorescence. We think it is unlikely that all of the flavonoids are true antagonists at P2X7, and in line with this, quercetin was extremely poor in blocking ATP-induced calcium influx in a fura-2 assay (Supplementary Fig. 3). The natural polyphenol baicalin has been shown to reduce P2X7 responses measured by a Fluo-4 assay in the mouse macrophage J774 cell line, and this was validated in an IL-1β secretion assay [38]. It is unknown whether this compound can also inhibit human P2X7.

One of the main findings from this work was the discovery of two new NAM compounds from the NCI library screen with micromolar potency at hP2X7: confertifolin and digallic acid (Fig. 1). Digallic acid is functionally related to gallic acid which has recently been suggested to have activity at P2X7 in animal studies of pain [39, 40], and there is evidence for inhibition of hP2X7 currents by electrophysiology and gallic acid had an IC_50_ value of 4.26 µM [40]. There are no previous reports of confertifolin having activity at P2X7.

The original aim of the study was to investigate potential PAMs at hP2X7. We previously identified that triterpenoid glycosides from *P. ginseng*, termed ginsenosides, were the most effective potentiators of P2X7. For this reason, we identified and tested glycosides found in some of the plant extracts exhibiting the largest potentiation. These included momordin Ic (from *K. scoparia*; not shown), tenuifolin (from *P. tenufolia*; not shown), dioscin, protodioscin, gracillin, and the aglycone diosgenin (from *D. nipponica*). Momordin Ic exhibited a high level of cytotoxicity, and tenuifolin had no effect on P2X7 responses (data not shown) and these were not explored any further. In contrast, dioscin, and to a lesser extent gracillin, could effectively potentiate P2X7 responses (Fig. 6). In silico docking studies (Supplementary Fig. 4) predicted polar interactions between the sugar moieties on dioscin with amino acid residues within our previously described putative positive allosteric site [18]. These sugars are likely to be essential for the potentiation of P2X7 responses, as the aglycone diosgenin could not potentiate responses. This is in keeping with our previous observations that the ginsenoside aglycone protopanaxadiol could not potentiate P2X7 functions [12]. Dioscin contains three sugars on carbon-3 (L-Rha L-Rha D-Glc) and has some similarities to saikosaponin A, which we previously demonstrated to show cytotoxic effects [28]. Dioscin has previously been shown to have neuroprotective effects and could reduce the expression of P2X7 and inflammasome components in rat PC12 cells [41]. This study did not report a cytotoxic action of dioscin but used much lower concentrations (57–230 nM) in their in vitro experiments [41]. The authors suggested dioscin may regulate P2X7/NLRP3 pathways but did not test dioscin directly on P2X7 functional responses. We have not investigated the dose dependency of the positive allosteric modulator action of dioscin at human P2X7, and at lower concentrations, the non-specific cytotoxic effect may be reduced. Further work also needs to be done to understand selectivity for P2X7. This molecule has a lot of similarities with the ginsenoside series of PAMs and could provide a new scaffold (a spirostanol) that could be chemically modified to make a series of novel PAMs utilising what we know about structure–activity relationship of glycosides at hP2X7 [28].

From the NCI natural product library, several compounds exhibited positive modulator activity at hP2X7 including scopafungin, a compound derived from *Streptomyces hygroscopicus* [42]. There is very little information regarding the biological activity of scopafungin, with most studies only reporting antibiotic and anti-fungal activity [42, 43] plus IL-1R1 inhibition [44]. It is interesting that this compound is from *Streptomyces*, the same genus of bacteria from which ivermectin and avermectins are produced [45]. Both ivermectin and several avermectin derivatives are positive allosteric modulators at P2X4 receptors [11], with ivermectin also being reported to have PAM activity at human P2X7 [46]. This finding may open up a new avenue for investigating bacterial metabolites as novel modulators of P2X receptors. For scopafungin, a detailed pharmacological characterisation into dose dependency and selectivity for P2X7 should now be undertaken. Chemical modifications could provide insight into the main groups important for activity. Polymyxin B is similar to scopafungin, having a cyclical structure and a hydrophobic tail moiety, and polymyxin B positive modulator activity on P2X7 was shown to be dependent on the hydrophobic tail [47]. Molecules such as scopafungin could be very interesting as they could offer dual activity for certain infectious diseases such as boosting host immune responses and, at the same time, providing anti-bacterial or anti-fungal activity.

The study of pharmacognosy has great potential when it comes to the identification of novel receptor compounds, but studies into the physiological effects of TCM plants in isolation are severely lacking. In the spirit of open access research and data sharing, we have provided all information from the TCM plant extracts classed as hits for follow-up studies to be performed. For scientists interested in natural products as new lead compounds, fractionation and further testing of these plant extracts will be essential to identify the individual compounds responsible for P2X7 inhibition or potentiation.

In conclusion, we have described a useful pipeline for screening plant extracts (or other natural product libraries) including interference assays. We have identified a number of potentially interesting compounds with activity at human P2X7 that could be further explored with medicinal chemistry approaches.

## Supplementary Information

Below is the link to the electronic supplementary material.

Supplementary file1 (DOCX 1.77 MB)

## Data Availability

Data is available upon specific request to the corresponding author.
