# Supplementary material for: Screening herbal and natural product libraries to aid discovery of novel allosteric modulators of human P2X7
Source: Purinergic Signal. 2024 Oct 22;21(2):365–79. doi: 10.1007/s11302-024-10055-6 (PMC12062478; doi:10.1007/s11302-024-10055-6)
Supplement: Supplementary file 1 — Supplementary file1 (DOCX 1.77 MB) [file 11302_2024_10055_MOESM1_ESM.docx]

Supplementary Information

**Supplementary Table 1: Natural product compounds with PAM activity at hP2X7**

| **Natural product identifier** | **P2X7 response**  **(% of control)** | **Immediate action upon injection** |
| --- | --- | --- |
| Predorine | 740.31 | X |
| Actinomycin | 188.44 |  |
| Julimycin II | 180.28 |  |
| Monoacetyl verrucan A | 170.65 | X |
| Folinic acid | 160.00 |  |
| Scopafungin | 159.83 |  |
| NSC785176 | 159.37 |  |
| Vanilline | 158.27 |  |
| Siomycin A | 157.10 | X |
| Nogamycin | 155.77 | X |
| NSC785157 | 152.97 |  |
| Vulpinic acid | 150.3 |  |
| Withanolide A | 149.93 |  |
| NSC785143 | 149.00 |  |
| NSC785173 | 148.84 |  |
| Lagosin | 148.84 |  |
| Perezone | 148.44 |  |
| Hexopyranuronic acid | 147.11 |  |
| NSC785186 | 146.91 |  |
| Picropodophyllin | 145.86 |  |
| Ossamycin | 145.55 |  |
| Curcumin | 144.58 |  |
| Crotonic acid | 142.15 |  |
| Medicarpin | 140.60 |  |
| Pentoxifylline | 140.48 |  |
| Compactin | 139.71 |  |
| Streptonigrin | 139.02 |  |
| Baccatin III | 136.25 |  |
| Levullinic acid | 134.82 |  |
| MLS003170979 | 129.73 |  |
| Menogaril | 129.28 |  |
| NSC785187 | 127.21 |  |
| Erythromycin cyclopentylproprionate | 126.67 |  |
| NSC785167 | 125.18 |  |

**Supplementary Table 2: List of potential negative allosteric modulators of hP2X7**

| **Natural product name** | **P2X7 response**  **(% of control)** |
| --- | --- |
| Digallic acid | 0 |
| Acronine | 1.05 |
| Confertifolin | 9.72 |
| Hydroberberine | 9.75 |
| Chaetochromin A | 12.08 |
| Aquamycin | 15.18 |
| Tetrahydroberberine | 26.9 |
| NSC332294 | 29.09 |
| Anthricin | 30.94 |
| Β-peltatin | 31.98 |
| Acetopapaverine | 34.92 |
| Sarnovid | 38.95 |
| Quadrone | 40.82 |
| Oleandrogenin | 42.33 |
| Borrelidin | 42.93 |
| Destrusin B | 46.65 |
| Tolypromycin Y | 47.05 |
| 3-desmethylcolchicine | 49.33 |
| NSC785158 | 49.59 |

**Supplementary Table 3: Plant extracts with inhibitory activity at hP2X7**

| **Plant name** | **Inhibits IL-1β secretion and YO-PRO-1 uptake** | **Inhibits ATP-induced cell death** | **Contains berberine or emodin** | **Hit** |
| --- | --- | --- | --- | --- |
| P suffruticosa | Yes | Yes |  | Y |
| A Oxyphylla | Yes | No |  |  |
| R rugosa | Yes | Yes |  | Y |
| C yanhusuo | Yes | Yes | Y |  |
| P multiflorum | Yes | Yes | Y |  |
| P nigrum | Yes | Yes |  | Y |
| S stoloniferum | No | Yes |  |  |
| P lactiflora | Yes | Yes |  | Y |
| R palmatum | Yes | No | Y |  |
| R cordifolia | Yes | No |  |  |
| J effusus | Yes | No |  |  |
| A dahurica | Yes | No |  |  |
| P veitchii | Yes | Yes |  | Y |
| A annua | Yes | Yes |  |  |
| P mume | Yes | Yes |  | Y |
| A paniculata | Yes | No |  |  |
| L chuanxiong | Yes | No |  |  |
| U rynchophylla | Yes | Yes |  | Y |
| A venetum | Yes | Yes |  | Y |
| A Pilosa | Yes | Yes |  | Y |
| C cristata | Yes | Yes |  | Y |
| L lucidus | Yes | Yes |  | Y |
| A tatrinowii | Yes | Yes | Y |  |
| I verum | Yes | Yes |  | Y |
| S dichotoma | Yes | Yes |  | Y |
| C medica | Yes | Yes |  | Y |
| A heterotropoide | Yes | Yes |  | Y |
| L lucidum | Yes | Yes |  | Y |
| E japonica | Yes | Yes |  | Y |
| B javanica | Yes | No |  |  |
| Z bungeanum | Yes | Yes |  | Y |
| A orientalis | Yes | Yes |  | Y |
| D longan | Yes | Yes |  | Y |
| P cocos | Yes | Yes |  | Y |
| S miltirrhiza | Yes | Yes | Y |  |
| V officinalis | Yes | No |  |  |
| S divaricata | Yes | Yes |  | Y |
| C indicum | Yes | Yes |  | Y |
| C chinesis | Yes | Yes | Y |  |

**Supplementary Table 4: Plant extracts with PAM activity at hP2X7**

| **Plant name** | **Potentiates IL-1β secretion** | **Potentiates ATP-induced cell death** | **Hit** |
| --- | --- | --- | --- |
| K scoparia | Yes | Yes | Y |
| D nipponica | Yes | Yes | Y |
| P grandiflorum | No | Yes |  |
| P tenufolia | Yes | Yes | Y |
| T farfara | Yes | No |  |
| C pinnatifida | No | No |  |
| P aviculare | Yes | Yes | Y |
| P notoginseng | Yes | No |  |
| S marianum | Yes | Yes | Y |
| C moriflorum | No | No |  |
| L gracile | No | Yes |  |
| P communis | No | No |  |
| I indigotica | Yes | Yes |  |
| P ginseng | No | Yes |  |
| L heterophyllus | No | Yes |  |
| H diffusa | No | Yes |  |
| E brevicornum | No | No |  |

**Supplementary Figure 1**

**A**


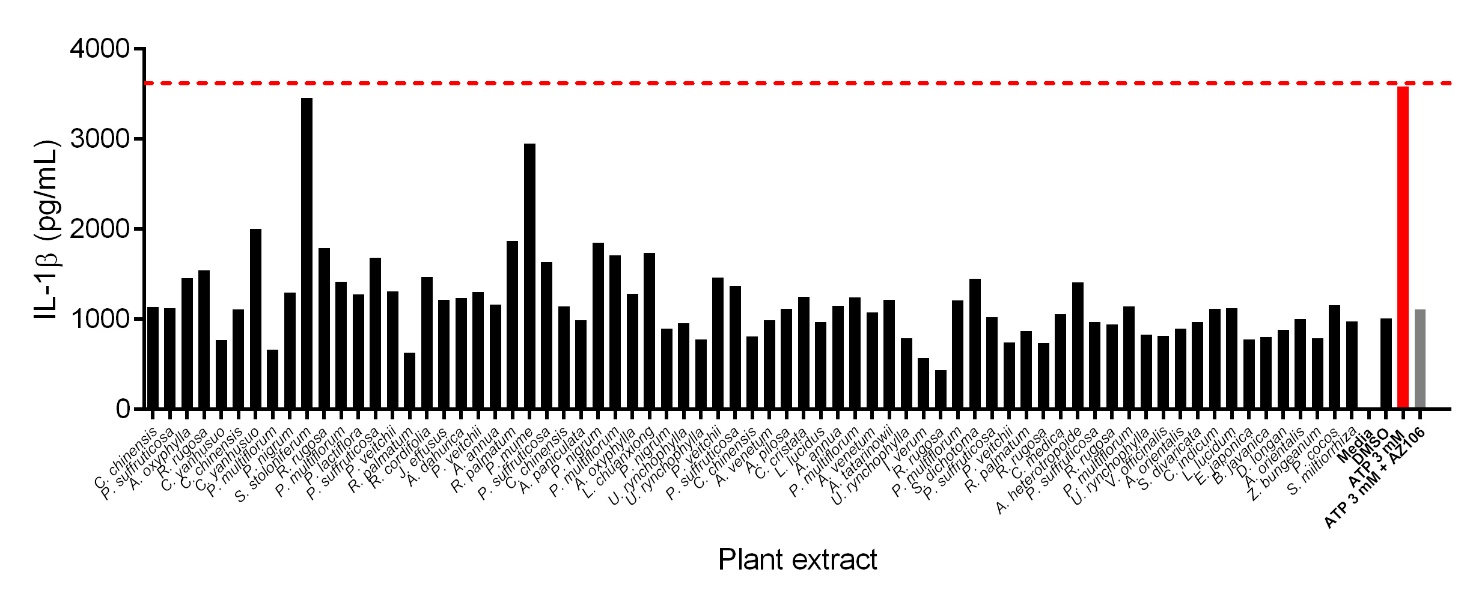


**B**


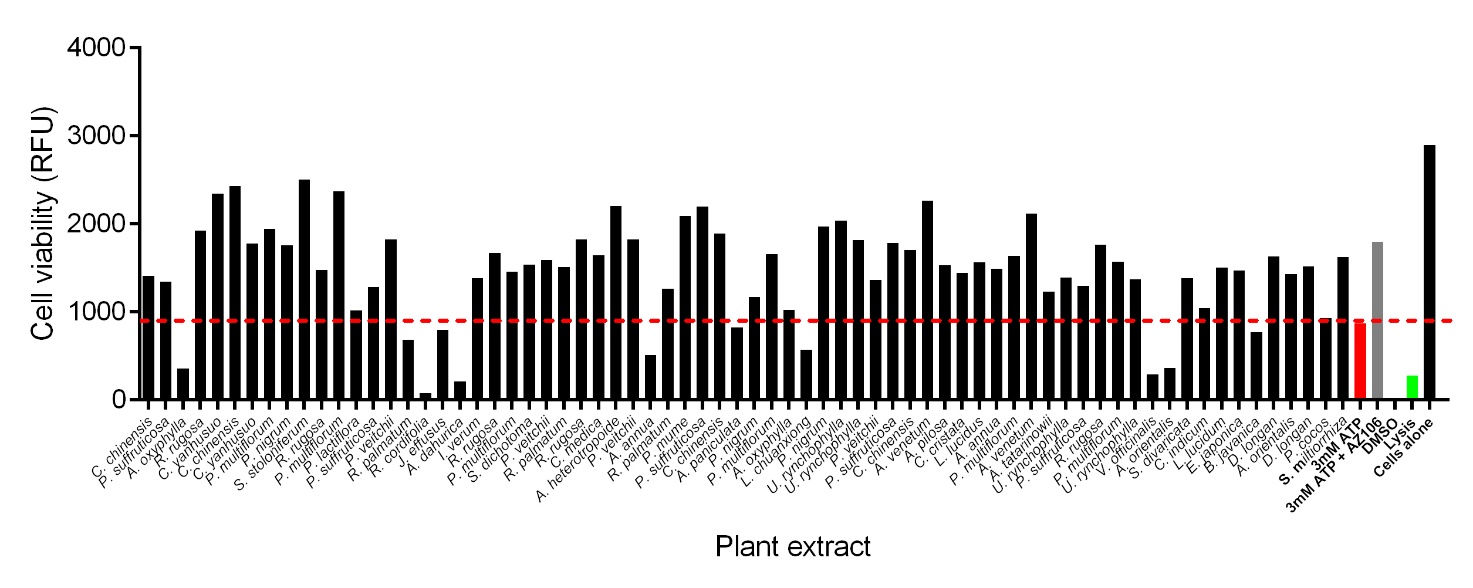


**Supplementary Figure 1. Extracts that inhibit P2X7 dye uptake responses can inhibit production/secretion of IL-1β and inhibit ATP-induced cell death. (A)** THP-1 cells were pre-treated with 100 ng/mL LPS and 30 µg/mL of TCM extract for 4 h, followed by a stimulation with 3 mM ATP for the final 30 min of incubation. IL-1β release was quantified and compared to the production of IL-1β in the absence of extracts. AZ10606120 (10 µM) was used as a positive control for P2X7 inhibition. **(B)** HEK-hP2X7 cells were treated with 3 mM ATP in the presence or absence of 30 µg/mL of TCM extract for 24 h prior to quantification of metabolically active cells using Alamar blue assay (resazurin 0.1mg/ml). AZ10606120 was used as a positive control for P2X7 inhibition, cells were lysed as a positive control for cell death. Data are representative of two technical replicates but one independent experiment due to the limited availability of the extracts.

**A**

**
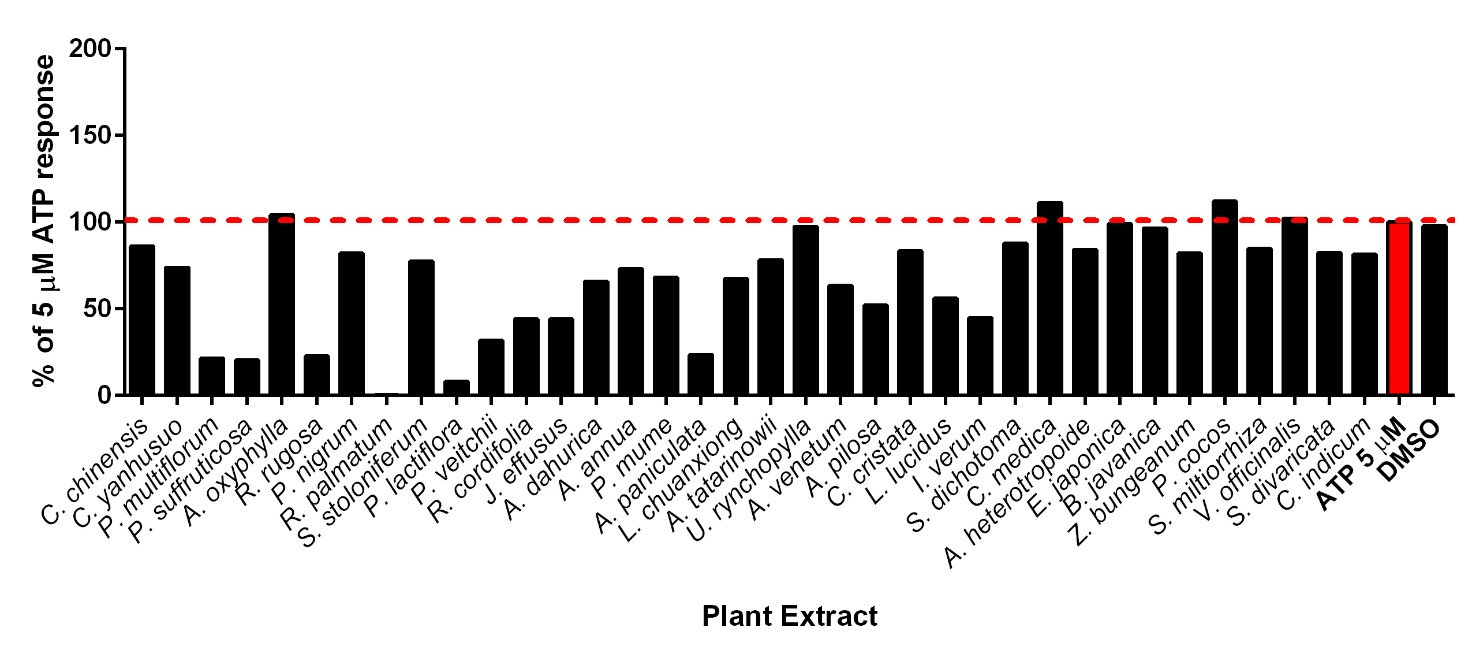
**

**
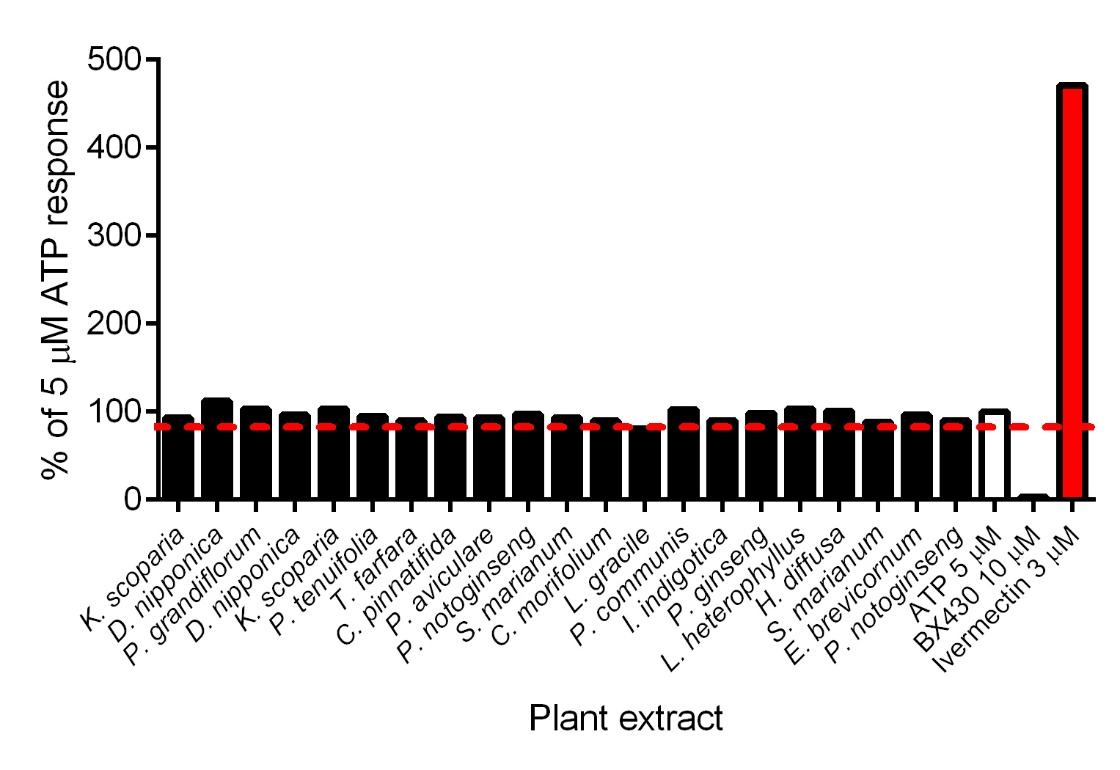
B**

**Supplementary Figure 2. The effect of plant extracts that have activity at hP2X7 against hP2X4. (A)** HEK-hP2X4 cells were pre-treated with 30 µg/mL of TCM extract for 60 seconds prior to the administration of 5 µM ATP. YO-PRO-1 uptake was measured for an additional 210 seconds and the YO-PRO-1 uptake between 200-300 seconds was quantified as a percentage of the ATP response in the absence of extracts. DMSO was used as a vehicle control, BX430 (10 µM) as a positive control. **(B)** As in (A) except Ivermectin (3 µM) was used as a positive control for potentiation and DMSO was used as a vehicle control. Data are representative of two technical replicates but one independent experiment due to the availability of the extracts.

**Supplementary Table 5: A list of compounds identified from a docking screen against the NAM site of hP2X7 and the associated plant species.**

| **Compound** | **Example Plant species** |
| --- | --- |
| Astragalin | *P. suffruticosa, R. rugosa, A. venetum* |
| Methyl gallate | *P. suffruticosa* |
| Luteolin | *R. rugosa* |
| Quercetin | *R. rugosa, A. venetum, I.verum, L.lucidum* |
| Palmitic acid | *R. rugosa,* |
| Lineolic acid | *R. rugosa,* |
| Kaempferol | *R. rugosa, P. mune, I.verum,* |
| Isoquercetin | *R. rugosa,* |
| Erythro-1-(4-hydroxy-3-methocyphenyl)propane-1,2-diol | *P. lactiflora,* |
| Paeonolide | *P. lactiflora* |
| Paoniflorigenone | *P. lactiflora* |
| Threo-1-(4-hydroxy-3-methoxyphenyl)propane-1,2-diol | *P. lactiflora* |
| Erythro-1-phenylpropane-1,2-diol | *P.lactiflora* |
| Citric acid | *P. mume* |
| 5-hydroxymethylfurfural | *P. mume* |
| Genistein | *P. mume* |
| Vallesiachotamine | *U. rhynchophylla* |
| 11-methoxyuncarine C | *U. rhynchophylla* |
| Corynoxine | *U. rhynchophylla* |
| Isobergapten | *C.medica, S.divaricata* |
| 5-hydroxy-7-methoxycoumarin | *C.medica* |
| Cycloartanol | *C.medica* |
| Citropen | *C.medica* |
| Atlantoflavone | *C.medica* |
| Bergamotine | *C.medica* |
| 2’4’6’-trimethoxyacetophenone | *C.medica* |
| Longifloriside | *L.lucidum* |
| 6’-O-cinnamoyl-8-epikingisidic acid | *L.lucidum* |
| 5-O-methylvisammioside | *S.divaricata* |
| Cassiachromone | *S.divaricata* |
| Prim-O-glucosylcimifugin | *S.divaricata* |
| Epi-eudesmol | *S.divaricata* |
| Psoralen | *S.divaricata* |
| Imperatorin | *S.divaricata* |
| Isoimperatorin | *S.divaricata* |
| Panaxynol | *S.divaricata* |
| Cimifugin 4’-O-beta-D-glucopyranoside | *S.divaricata* |
| 1,2,3,19-tetrahydroxy-12-ursen-28-oic acid | *A.pilosa,* |
| Biondinin C | *I.verum* |
| Dunnianol | *I.verum,* |
| Isodunnianol | *I.verum,* |
| Isomagnolone | *I.verum,* |
| Dehydrodiconiferyl alcohol | *I.verum,* |
| Lariciresinol dimethyl ester | *I.verum,* |
| 5-O-caffeoylshikimic acid | *I.verum* |
| 3-O-caffeoylshikimic acid | *I.verum* |
| 4-O-caffeoylshikimic acid | *I.verum* |
| Scopoletin | *A. venetum, A.annua* |
| Agrimonolide 6-O-glucoside | *A.pilosa,* |
| Luteolin-7 | 1. *pilosa* |
| Luteolin-6 | *A.pilosa,* |
| Cosmosiin | *A.pilosa,* |
| (+)-Taxifolin | 1. *pilosa* |
| Bergapten | *C.medica S.divaricata* |
| Narirutin | *A. venetum* |
| 5,7-dihydroxy-2-isopropylchromone | *A.pilosa,* |
| 3,6,19-tetrahydroxy-23-oxo-12-ursen-28-oic acid | *U. rhynchophylla* |
| Emodin | *R. palmatum, P.multiflorum, A.tatarinowii* |
| Berberine | *C.chinesis, C.yanhusuo* |

**Supplementary Figure 3**


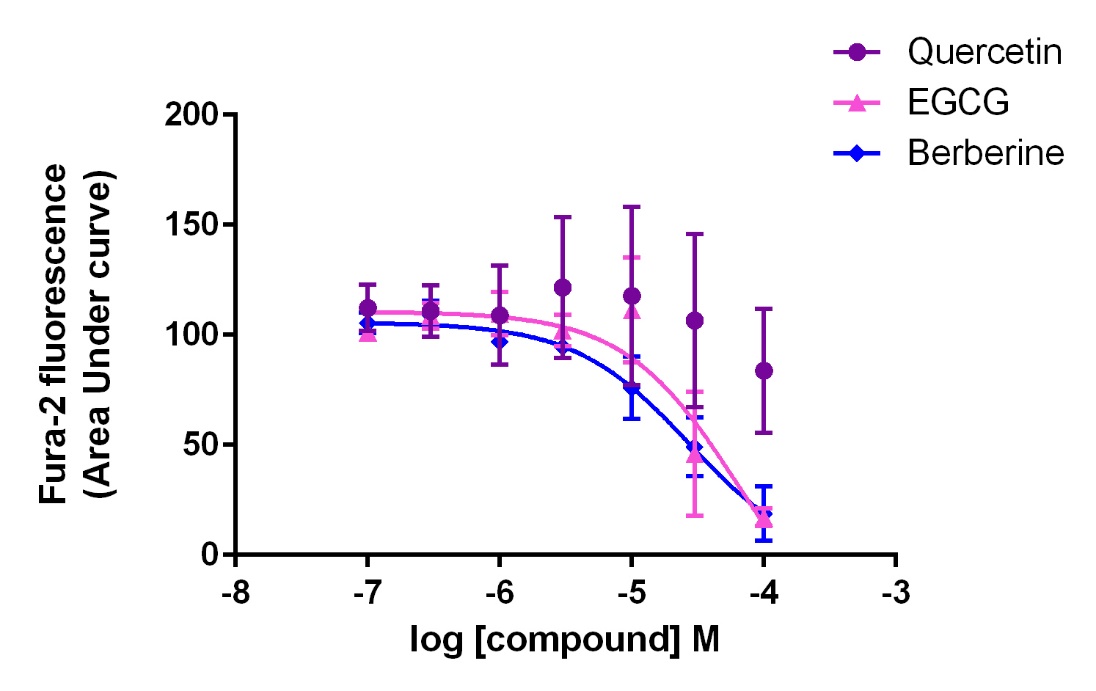


**Supplementary Figure 3: Validation of P2X7 antagonist activity using a fura-2 assay.** HEK-hP2X7 cells were loaded with fura-2AM (2µM) to measure intracellular calcium responses to ATP. Cells were loaded for 45 minutes at 37˚C and then pre-treated with various concentrations of test compounds for 10 minutes. For full methodological details please see [1]. Responses to 1mM ATP were assessed over 300 seconds using a Flexstation 3 plate reader. Ratiometric fura-2 data was converted to area under curve.


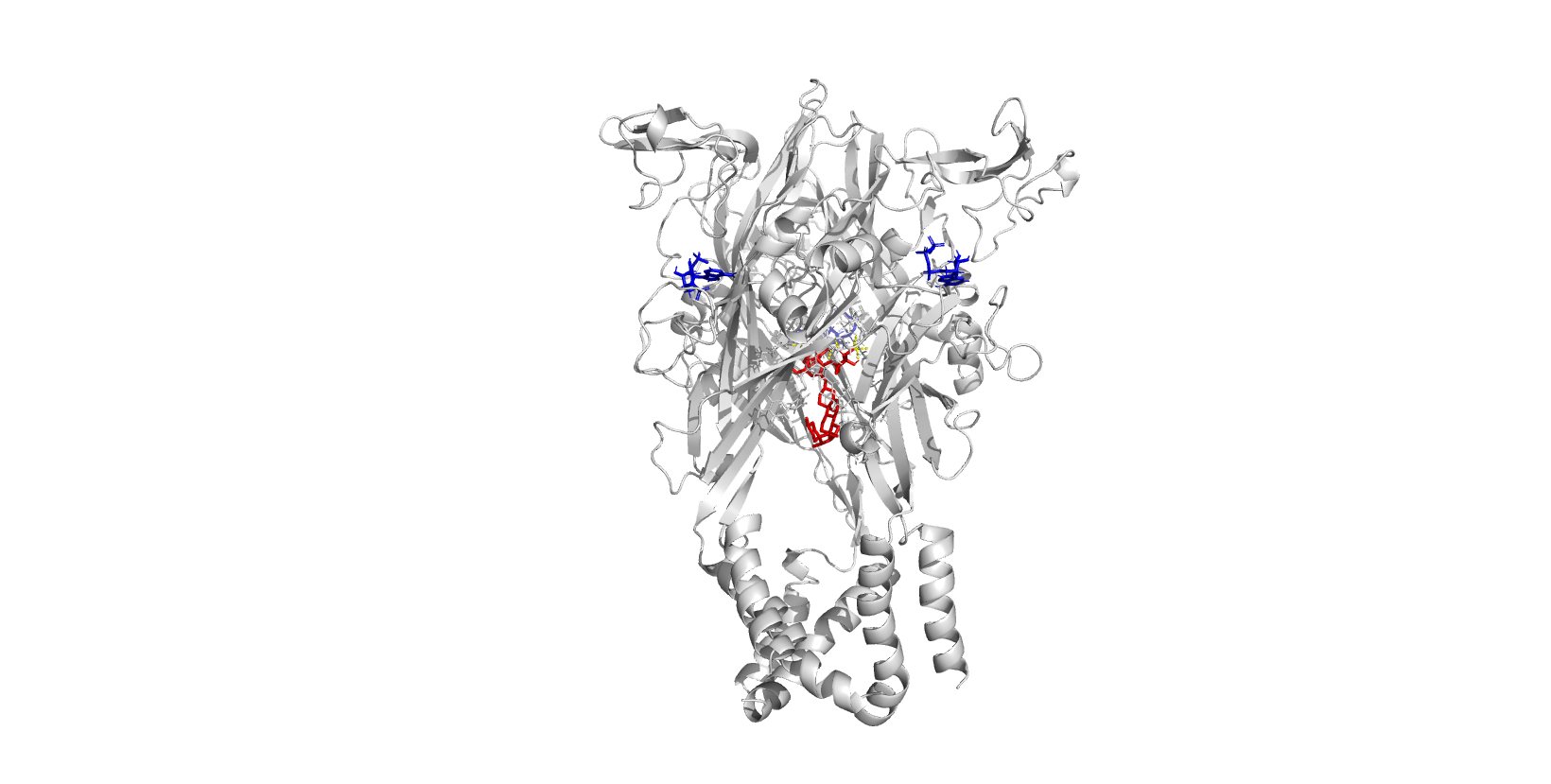


**B**

**A**


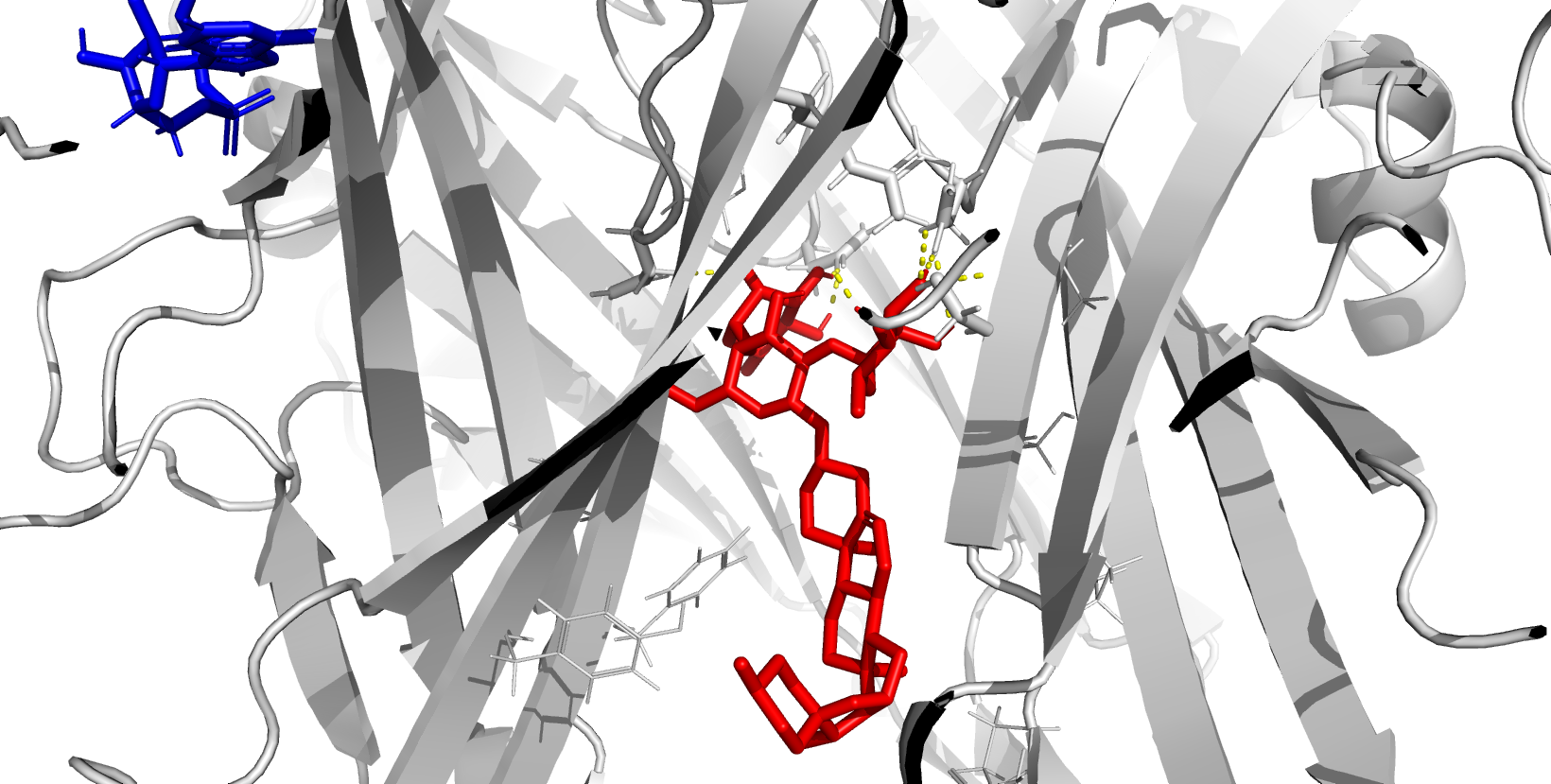


**N100**

**N100**

**R316**

**S60**

**N100**

**Supplementary Figure 4:** Computational docking of dioscin to a homology model of human P2X7. (A) Dioscin is shown docked into the central vestibule of P2X7 (open state). Three molecules of ATP as shown in the orthosteric pocket. (B) A close-up image of dioscin in the central vestibule pocket showing polar contacts with three N100 residues from the three subunits, plus R316 and S60.

**References**

[1] Dhuna K., et al., *Ginsenosides act as positive modulators of P2X4 receptors.* Molecular Pharmacology, 2019, 95:210-221.
